# Context-Aware Multi-Scale Aggregation Network for Congested Crowd Counting

**DOI:** 10.3390/s22093233

**Published:** 2022-04-22

**Authors:** Liangjun Huang, Shihui Shen, Luning Zhu, Qingxuan Shi, Jianwei Zhang

**Affiliations:** 1 School of Computer Science and Information Engineering, Shanghai Institute of Technology, Shanghai 201418, China; 206142102@mail.sit.edu.cn (S.S.); 196141112@mail.sit.edu.cn (L.Z.); 2 School of Cyber Security and Computer, Hebei University, Baoding 071000, China; shiqingxuan@hbu.edu.cn; 3 Department of Informatics, University of Hamburg, D-20146 Hamburg, Germany; zhang@informatik.uni-hamburg.de

**Keywords:** dense crowd counting, multi-scale feature learning, convolutional neural network

## Abstract

In this paper, we propose a context-aware multi-scale aggregation network named CMSNet for dense crowd counting, which effectively uses contextual information and multi-scale information to conduct crowd density estimation. To achieve this, a context-aware multi-scale aggregation module (CMSM) is designed. Specifically, CMSM consists of a multi-scale aggregation module (MSAM) and a context-aware module (CAM). The MSAM is used to obtain multi-scale crowd features. The CAM is used to enhance the extracted multi-scale crowd feature with more context information to efficiently recognize crowds. We conduct extensive experiments on three challenging datasets, i.e., ShanghaiTech, UCF_CC_50, and UCF-QNRF, and the results showed that our model yielded compelling performance against the other state-of-the-art methods, which demonstrate the effectiveness of our method for congested crowd counting.

## 1. Introduction

The main aim of the crowd-counting task is to calculate the number of people present in an image or video. This research topic has been receiving much attention due to its enormous value in practical applications such as video surveillance [1,2], public safety [3,4], human behavior analysis [5], and traffic control [6].

The goal of crowd-counting tasks has gradually developed from detecting individual persons in a single image to estimating crowd density. Image or video scenes are often severely occluded, and crowd scales vary widely, making the task of accurately counting crowds difficult, especially for dense scenes. Inspired by the great success of convolutional neural networks (CNNs) in computer vision tasks, researchers have recently proposed many crowd counting methods based on CNNs [7,8]. These methods attempt to perform the crowd counting task by utilizing multi-scale feature learning [9,10], multi-task learning [11,12], and attention mechanisms [13,14]. Most methods do not perform well in scenes with dramatic crowd scale changes and complex environments. Therefore, designing a network that can efficiently model large-scale head changes and at the same time enhance the ability of crowd recognition for complex scenes is critical to improving crowd counting accuracy.

**Figure 1:**
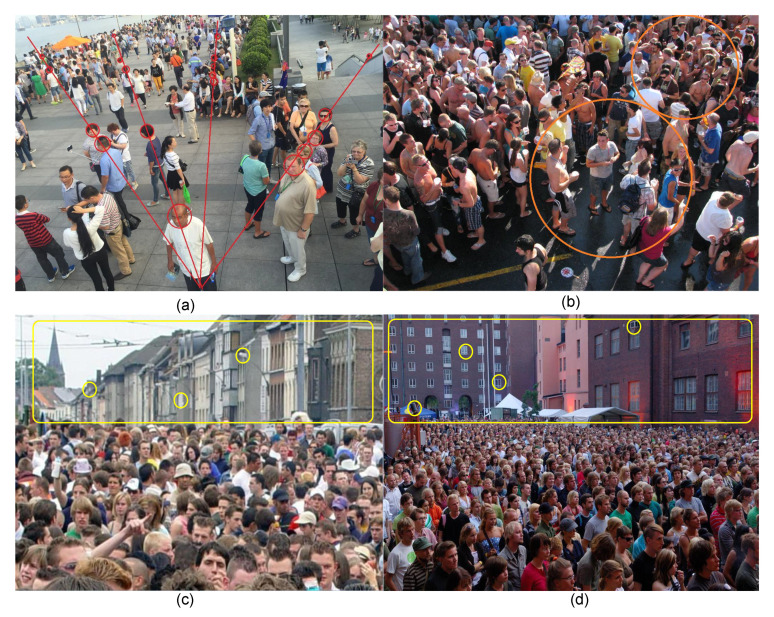
Visual results of different crowd scenes. Image (**a**) depicts the scale of the distance from the camera. Image (**b**) shows the head-blocking phenomenon caused by crowd congestion. Images (**c**,**d**) show the effects of objects resembling a human head in the background.

The scale and distribution of a crowd vary in an image. On the one hand, because the distances between the crowd and the camera vary, the people at a longer field of view along a straight line appear relatively crowded, and the head scales are different (as shown in Figure 1a). On the other hand, some areas are overcrowded, and the crowd is close together, which leads to mutual occlusion between human heads (as shown in Figure 1b). In addition to the crowd distribution, the influence of background objects presents a challenge. Since the image or video is captured from different and changing environments, the content presented in the image or video contains crowd and non-crowd information. Background objects similar in shape to human heads can be incorrectly recognized by the counting network as “human heads” (yellow circles in Figure 1c,d). In previous works, multi-column convolution [15,16] and dilated convolution [17,18] methods were usually used to extract features for different scales, but these methods do not make full use of the representations of crowd features in the context information for scale extraction. Moreover, their networks entail a large number of parameters and floating-point calculations.

To solve the above challenges, we propose a context-aware multi-scale aggregation network named CMSNet for dense crowd counting. Its purpose is to address the challenge of inconsistent head scales in crowded scenes and enhance the ability of the method to recognize the features of crowds in an image. The MSAM adopts different dilation rates to extract multi-scale features of the feature maps in each channel and then fuses them. In addition, the CAM is used to enhance the crowd feature information of the initial feature map in each channel. Then, CMSNet aggregates the multi-scale aggregation information and the enhanced information for the crowd to generate a density map. As the number of channels in the network decreases and the feature map is enlarged step by step, a density map of the original size is generated to predict the number of people. The contributions of this paper are summarized as follows:

1. We propose a new MSAM. Different dilation rates are used to obtain multi-scale feature information. In addition to each multi-scale sampling branch, a branch of the global receptive field (GRF) is added to help the other multi-scale branch sample features more accurately.
2. We propose a new CAM, which uses an attention mechanism to identify the features of the crowd information in an image by relying on the context information.
3. We propose a novel context-aware multi-scale aggregation network named CMSNet for dense crowd counting, which utilizes a weighted attention method to strengthen the expression of crowd information and a multi-scale sampling method to obtain information at different scales, thus improving the counting accuracy.

Compared with other state-of-the-art methods presented in recent years, our proposed method shows validity and competitiveness on three challenging crowd datasets, i.e., ShanghaiTech, UCF_CC_50, and UCF-QNRF.

The rest of the paper is organized as follows. First, we review traditional crowd-counting methods and counting methods based on convolutional neural networks (CNNs) in Section 2. The multi-scale feature learning methods are briefly introduced. Then, we introduce the method proposed in this paper in Section 3. We present the experimental results on the datasets and an ablation analysis in Section 4. Finally, we draw conclusions in Section 5.

## 2. Related Work

### 2.1. Traditional Methods

Traditional crowd-counting methods use hand-crafted features for modelling crowds, and leverage machine learning [19] methods to recognize crowds. These include detection-based methods [20,21], regression-based methods [22,23], and density map-based methods [24,25]. Early detection methods mainly designed object detectors to recognize pedestrians in a crowd [26] and they obtained the number of pedestrians in a scene by counting the number of detection frames. However, such methods usually have low counting accuracy due to the high pedestrian density in a crowd and mutual occlusion among pedestrians. To perform crowd estimation in high-density cases, researchers proposed a regression-based method [27]. The main idea of this method is to extract the crowd features and train the regressor to construct a nonlinear mapping between the crowd image and the number of people. Although this method has better detection accuracy than the early detection methods, it does not provide clear results for the location distribution information of pedestrians in the crowd. Finally, to obtain the location information of pedestrians in a crowd, Lemptisky et al. [25] first adopted a method based on density estimation. This method mainly learns a linear mapping relationship between local features and the corresponding density maps. However, the acquisition of the density map depends on manual extraction, and different scenes must be manually extracted again, which greatly limits the generalization ability of the algorithm.

### 2.2. CNN-Based Methods

Due to the strong feature-learning ability of CNNs, many counting methods based on CNNs have appeared for crowd counting tasks. Zhang et al. [28] first adopted the CNN method to design a network model for crowd density estimation. With the emergence of various network structures, the accuracy of the crowd counting task has also been improved. Li et al. [17] first applied dilated convolution to crowd counting and greatly improved the calculation accuracy. Shen et al. [11] used the concept of generative adversarial nets (GAN) [29] to generate density maps using the U-NET structure [30] and used the consistency of calculation between subimages and general images to improve the calculation accuracy. The accurate recognition of multi-scale information and the accurate representation of crowd feature information have attracted the attention of many scholars.

### 2.3. Multi-Scale Feature Learning

This method is designed to use multi-scale features or contextual information to address the challenge of head scale variation in a crowd. The multi-column convolutional neural network (MCNN) proposed by Zhang et al. [15] used multi-scale filter kernels to extract features with receptive fields of different sizes. Similarly, Sam et al. [16] proposed Switching-CNN, which used a switching classifier to select the best classifier from a density-class classification pool for crowd estimation. The method for generating high-quality crowd density maps using contextual pyramid CNN (CP-CNN) proposed by Sindagi et al. [31] generated high-quality density maps and captured multi-scale information by combining prior information from global and local contexts. In addition, Sindagi and Patel et al. [32] proposed a multi-level bottom-top and top-bottom feature fusion network (MBTTBF), which was elaborately designed to combine multi-scale information with multiple shallow and deep features. Liu et al. [33] designed an attention map generator (AMG) module for ADCrowdNet that adopted multi-scale acquisition methods for different channel dimensions to improve its ability to acquire different scale information. Zhang et al. [34] proposed the attentional neural field (ANF), which combined a conditional random field and a nonlocal attentional mechanism to capture multi-scale features and long-range correlation, enhancing the ability of the network to work with large-scale changes. Recently, Yang et al. [35] proposed the use of dual-stream ResBlock matching features of different scales so that the network could automatically learn and aggregate matching scores of different scales to improve the recognition accuracy of different objects. In contrast to the above methods, the network presented in this paper uses GRF information to help express the features of each scale, fuses multi-scale information to generate a density map, and estimates the number of people. Inspired by Modolo et al. [36], we use a separate context-aware branch in this network to enhance the feature information identification for the crowd, in which the attention mechanism is the main measure of this branch. This method utilizes the visual attention mechanism to make the counting network consciously focus on useful information to improve the counting performance.

## 3. Proposed Method

### 3.1. Overview

**Figure 2:**
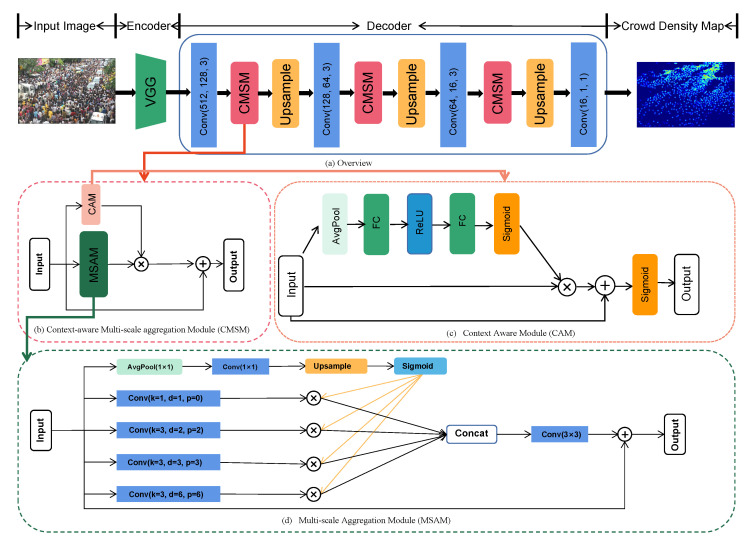
Overview of CMSNet. An image is received by the encoder and converted into deep features, and then the deep features are decoded by the decoder into a density map for crowd counting. The decoder is composed of three context-aware multi-scale aggregation modules (CMSMs), and the CMSMs are connected in parallel by a CAM and an MSAM to generate feature maps at each channel level.

Similar to the current mainstream methods [15,16,17], we treat the crowd-counting problem as a pixel-level regression task. CMSNet is divided into an encoder and a decoder, as shown in Figure 2a. The encoder uses the first 10 convolutional layers of VGG16 [37] to gradually reduce the image size and extract deep features. As the main design of this study, the decoder adopts multi-scale fusion and feature enhancement methods to decode and reconstruct the features obtained by the encoder. Features are obtained through convolutional layer narrowing channels, and the features are input into the CMSM (Figure 2b) to accurately obtain multi-scale feature maps. Then, the enhanced feature map is upsampled by a factor of 2 to obtain the enlarged image. After three repetitions of this step, a 16-channel feature map is obtained. The final predicted density map is obtained through a 1×1 convolutional layer. The overall operation structure of the network can be described as extracting the primary features from the deep features by inputting an image $I_{i}$ and using Equation (1),

(1) $$f_{i}=F_{\mathrm{VGG}}\left(I_{i}\right),$$

where $F_{\mathrm{VGG}}$ represents the encoder, which is used to extract the deep features. The extracted features are then fed into the decoding network to produce a density map for prediction $G_{i}$,

(2) $$G_{i}=F_{\mathrm{decoder}}\left(f_{i}\right),$$

where $F_{\mathrm{decoder}}$ represents the main design of this paper, the decoder, which is mainly composed of the CMSM. The CMSM is used to fuse the enhanced crowd feature information and multi-scale information.

### 3.2. Context-Aware Multi-Scale Aggregation Module

In the crowd-counting task, enhancing the expression of crowd information is an essential method. The common method is to use an attention mechanism [38,39] to assign different weight values to channel or spatial features and then apply the weight values to the features for learning. In this study, we use the CMSM designed as the parallel combination of a CAM (Figure 2c) and an MSAM (Figure 2d). The CAM adopts a scaling and stretching feature mechanism, uses the residual connection to enhance the feature recognition for the crowd and surrounding information, and performs different feature weighting processing on the feature maps generated by the convolutional layer of the decoder network to enhance the crowd feature information. Different dilation rates are used to sample feature information at different scales in the MSAM, and the branch with the GRF information is applied to the other four multi-scale branches to assist multi-scale information learning (as shown in the orange flow line in Figure 2b). Then, the feature information obtained by the CAM and the MSAM is merged to obtain the feature in the current channel. The specific formula is as follows:

(3) $$f_{i}^{\mathrm{CAM}}=F_{\mathrm{CAM}}\left(f_{i}\right),$$

(4) $$f_{i}^{\mathrm{MSAM}}=F_{\mathrm{MSAM}}\left(f_{i}\right),$$

(5) $$f_{i}^{{}^{\prime }}=f_{i}^{\mathrm{CAM}}*f_{i}^{\mathrm{MSAM}}+f_{i},$$

where $f_{i}$ represents the input feature map of the *i*-th image in the current channel, $f_{i}^{\mathrm{MSAM}}$ represents the feature of the *i*-th image in the current channel processed by the MSAM, $f_{i}^{\mathrm{CAM}}$ represents the weight features of the *i*-th image in the current channel after the CAM, and $F_{\mathrm{CAM}}$ and $F_{\mathrm{MSAM}}$ represent the CAM and MSAM, respectively. A residual connection is adopted to help stabilize the network, prevent gradient disappearance and better learn the features in Equation (5). ∗ represents the multiplication of matrix elements. Finally, the CMSM inputs the processed feature map into the next stage.

#### 3.2.1. Multi-Scale Aggregation Module

To explore an appropriate multi-scale structure for feature recognition and learning of the head region in the feature map, this study adopts a multi-scale aggregation method designed similarly to atrous spatial pyramid pooling (ASPP) [40]. For the crowd in an image, each head is different in size. The dilation rates should consider the “small” heads in the edge areas as well as the “large” heads near the camera. Compared with that of [40], the structure designed in this study adopts more reasonable dilation rates and padding rates for different head sizes in an image, as shown on the bottom line of Figure 2d. In crowded scenarios, dilation rates and padding rates are set to be relatively small to better measure the number of heads. In contrast, larger dilation rates are used to detect heads close to the camera.

The network has an added branch that enables the MSAM to obtain the GRF. The GRF first compresses all information into a feature map with a size of 1×1, then restores it to the original size and sets a probability within (0,1) to improve the multi-scale detection capability of the other four branches. Finally, all scale information is fused and output. With the help of the GRF, the other four dilated branches can better learn different feature representations. The details are shown in the following formulas:

(6) $$\begin{array}{c} P_{i}=\mathrm{Sigmoid}\left(\mathrm{Upsample}\left({\mathrm{Conv}}_{1}\left(F_{\mathrm{avg}}\left(f_{i}\right)\right)\right)\right), \end{array}$$

(7) $$f_{i,j}=P_{i}*{\mathrm{Conv}}_{i,j}^{d},d=1,2,3,6,$$

(8) $$f_{i}^{\mathrm{MSAM}}={\mathrm{Conv}}_{3}\left(\mathrm{Concat}\left(f_{i,j}\right)\right)+f_{i},$$

where $f_{i}$ represents the input feature map of the *i*-th image in the current channel. $F_{\mathrm{avg}}$ compresses the feature map to features of size (1, 1). After a 1×1 convolution operation on layer ${\mathrm{Conv}}_{1}$, Upsample is used to restore the feature map to its original size, and Sigmoid is used to set it to the probability interval of (0, 1) to obtain $P_{i}$ and ensure that it has a GRF. *d* in ${\mathrm{Conv}}_{i,j}^{d}$ represents the dilation rate, and *i* and *j* represent the *i*-th image and *j*-th branch of the current channel, respectively (j=1,2,3,4). ${\mathrm{Conv}}_{3}$ is represented by a 3×3 convolution operation. $f_{i}^{\mathrm{MSAM}}$ is the final feature generated by the MSAM of the current channel.

#### 3.2.2. Context-Aware Module

In this paper, the CAM performs enhanced feature recognition on the feature maps received by each channel of the decoder to improve the ability to recognize the crowd. This module adopts an attention mechanism similar to that of SENet [38] and adds a residual connection on this basis to assist the network in recognizing crowd features by relying on context information. Then, the module uses Sigmoid to set probabilities within (0, 1) to perform preliminary overall crowd recognition for the initial feature map in each channel (as shown on the right in Figure 2c). The specific operation is shown in the following formula:

(9) $$\begin{array}{c} P_{i}^{\mathrm{CAM}}=\mathrm{Sigmoid}\left(\mathrm{Sigmoid}\left(W_{\mathrm{FC}}^{2}\left(\mathrm{ReLU}\left(W_{\mathrm{FC}}^{1}\left(F_{\mathrm{avg}}\left(f_{i}\right)\right)\right)\right)\right)+f_{i}\right), \end{array}$$

where $W_{\mathrm{FC}}^{1}$ and $W_{\mathrm{FC}}^{2}$ represent the first and second fully connected layers, respectively. Through the residual connection, CAM can stabilize the values in the feature map and increase the weight from the channel level to help the network use the surrounding information to learn the feature information of the crowd.

### 3.3. Density Map Generation and Loss Function

Similar to mainstream methods [15,16,41,42], the generation method of the density map adopts an adaptive Gaussian kernel [25]; that is, each head anchor point in the image is processed by the Gaussian kernel function:

(10) $$F\left(x\right)=\sum_{i=1}^{N}\delta \left(x-x_{i}\right)*G_{\sigma_{i}},\sigma_{i}=\beta {\bar{d}}^{i},$$

where $G_{\sigma_{i}}$ represents the two-dimensional standard Gaussian kernel function, δ() denotes the Dirac delta function, σ represents the standard deviation, and *N* is the total number of people in image $I_{i}$. β is a constant, and ${\bar{d}}^{i}$ is the diameter of a head in the image, which is the average distance between the *k* nearest-neighbor heads (k=7). Similar to Zhang et al. [15], this study sets β as 0.3. The processed images and the corresponding ground-truth density maps are reversed, mirrored, and cropped to expand the existing dataset. In this way, the mapping between the input image $I_{i}$ and the corresponding crowd density map F(x) can be obtained.

Moreover, the loss function of the network uses L2 loss to measure the difference between the output density map and the corresponding ground truth. The loss function is defined as follows:

(11) $$L\left(\lambda \right)=\frac{1}{2N}\sum_{i=1}^{N}{∥\hat{y}\left(I_{i};\lambda \right)-y_{i}∥}_{2}^{2},$$

where λ represents the learning parameter of the crowd-counting network and $\hat{y}\left(I_{i};\lambda \right)$ is the output of the crowd-counting network. $y_{i}$ is the ground truth. *N* represents the number of training images.

## 4. Experiments

### 4.1. Datasets

**ShanghaiTech** [15]: This dataset consists of two parts, ShanghaiTech Part_A (SHHA) and ShanghaiTech Part_B (SHHB). SHHA contains 482 crowd images from Internet searches. 300 images were used for the training set and 182 for the test set. In this dataset, the population size ranges from 33 to 3139 people, which is a large scope and can provide a good test of the network’s ability to handle variations in the population size. SHHB includes 716 crowd images taken from Shanghai’s busy streets and scenic spots. A total of 400 images were used for the training set, and 316 images were used for the test set.
**UCF_CC_50** [22]: This dataset contains many images of very crowded scenes, mostly from FLICKR. The number of images in this dataset is very limited (only 50 images), but the variation range of the number of people is very large (the number of people in one image reaches 4543), which brings great challenges for training and testing of the network. Similar to other mainstream test methods, we use 5-fold cross-validation for evaluation.
**UCF-QNRF** [43]: This dataset contains 1535 crowd images, of which 1201 images were used for training and 334 for testing. In addition, the number of people in the images in this dataset varies from 49 to 12,865, so it is a great option for testing the performance of the network.

Table 1 is the summary information for these three datasets.

### 4.2. Evaluation Metrics and Implementation Details

In this study, as the measurement standards for the density maps generated by the network compared to the ground truth maps, we adopted the mean absolute error (MAE) and mean square error (MSE) evaluation standards, consistent with mainstream methods, to measure the evaluation performance of the network.

(12) $$\mathrm{MAE}=\frac{1}{N}\sum_{i=1}^{N}\left|{\hat{y}}_{i}-y_{i}\right|,$$

(13) $$\mathrm{MSE}=\sqrt{\frac{1}{N}\sum_{i=1}^{N}{\left(y_{i}-{\hat{y}}_{i}\right)}^{2}},$$

where *N* represents the number of test images, $y_{i}$ represents the ground truth maps, and ${\hat{y}}_{i}$ represents the predicted density maps.

We used the first 10 convolutional layers of VGG16 pretrained in the ImageNet competition as the feature extractor, namely, the encoder. The initial learning rate was set to $7\times 10^{-6}$. Meanwhile, Adam with momentum was selected as the optimizer. All experiments were performed on a PC with a single GeForce RTX 2080Ti GPU and an Intel(R) I9-9900K CPU. The data preprocessing was set according to the C-3 framework structure [44]. In addition, the batch size of the ShanghaiTech dataset was set to 8, and those of the remaining datasets were set to 1. For simplicity, we used SHHA&B to represent the ShanghaiTech_part A&B dataset in our experiments.

### 4.3. Ablation Study

This section examines the ablation experiments between network modules to better prove the rationality and effectiveness of the network design presented in this paper.

#### 4.3.1. Ablation for the MSAM

**Figure 3:**
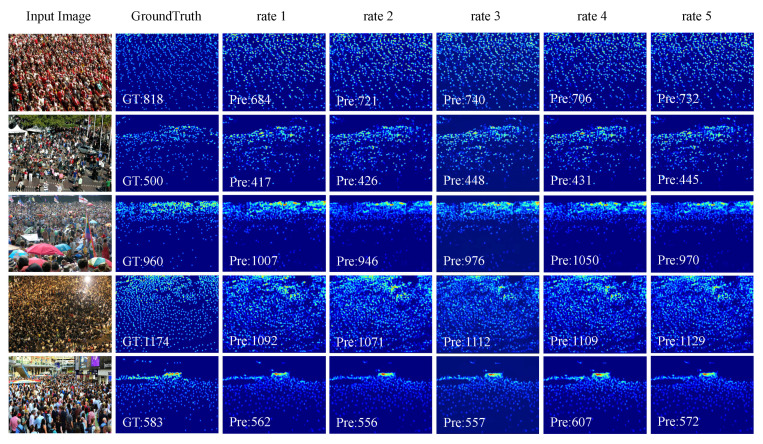
Visual results of the MSAM with different dilation rates on SHHA. From left to right: input image, ground truth, {1,2,3,4}, {1,2,3,5}, {1,2,3,8}, {1,4,7,9}, {1,2,3,6}.

We explored some sampling methods for the MSAM. Due to the different sizes of human heads in an image, the MSAM should handle extremely large human heads, very small human heads, and partial human heads blocked by crowd congestion. In terms of scale setting, a small dilation rate of “1–3” should still be taken for the subject as the sampling bottom line. Additionally, for heads with larger scales, a larger dilation rate in a single column should be used to obtain information on them. We also explored stepped dilation rates (1, 4, 7, 9) to obtain information on different head scales. However, in the crowd-counting task, the images of existing datasets contain a very wide range of head scales. Therefore, small-scale human heads were taken as the main body in the image, so if the dilation rate was set too large, then the sampling information would not be accurate. This can also be seen from the experimental results in Table 2 and Figure 3. In addition, when the number of module parameters was increased (as seen from Table 2), the counting performance did not improve, but decreased. Therefore, (1, 2, 3, 6) were chosen as the default parameters of the network.

**Figure 4:**
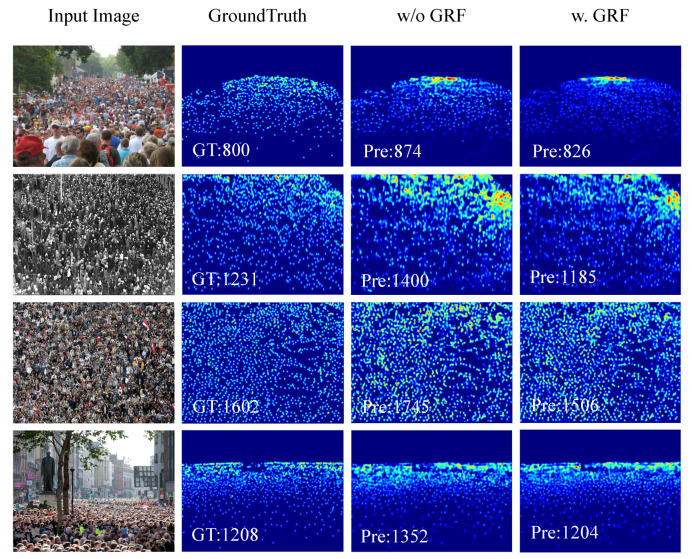
Visual results of effect of the GRF. From left to right: input image, ground truth, w/o GRF and w. GRF.

In addition, a GRF branch was added separately to the MSAM of this network. This branch helped every other scale branch better use the surrounding information to sample the crowd feature information. The experimental results are shown in Table 3 and Figure 4. The experimental results show that the GRF branch was effective in detecting multi-scale features.

We also explored the effect of the residual connection on this module. The experimental results are shown in Table 4. It can be proved that the residual connection can be used to provide the counting network with more feature information from before and after regression learning of crowd information to obtain better results.

#### 4.3.2. Ablation for the CAM

In the CAM, this network adopts the structure of SENet [38], with some parts modified according to the objectives and tasks of our research. In the dataset images, there are large-scale variations in the crowd features. Obtaining features of different crowd scales in the training stage is difficult. We added a residual connection to the [38] structure to help the network learn the features of the crowd context while maintaining the consistency of the network learning before and after. The specific experimental results are shown in Table 5.

The experimental results show that the increased residual connection can effectively help the network learn crowd features from context.

#### 4.3.3. Ablation for the CMSM

**Figure 5:**
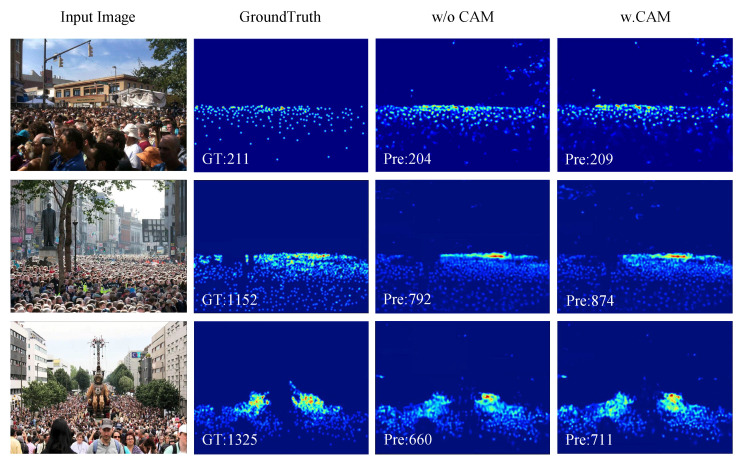
Ablation study of the CAM on SHHA. From left to right: input image, ground truth, result of w/o CAM, and result of w. CAM.

**Figure 6:**
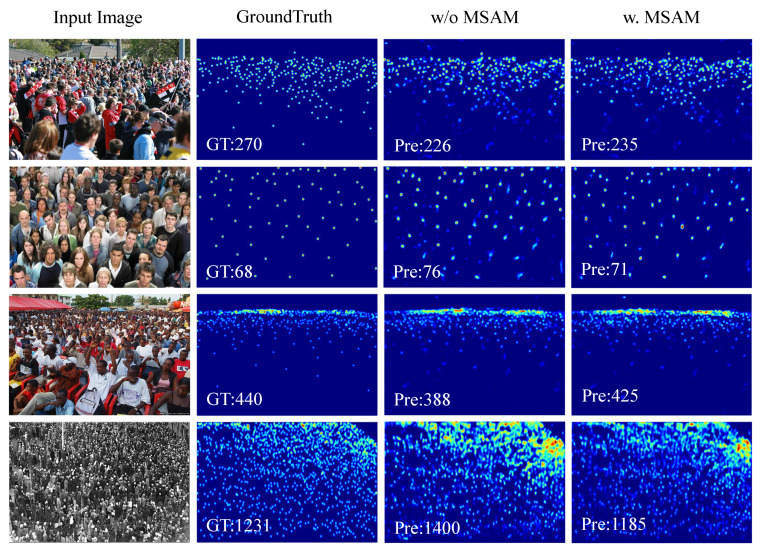
Ablation study of the MSAM on SHHA. From left to right: input image, ground truth, result of w/o MSAM, and result of w. MSAM.

In this part, the network separates the MSAM and the CAM from the CMSM. Then, the corresponding ablation experiments verify whether these two modules play a role. The experimental results are shown in Table 6, Figure 5 and Figure 6. In the third row of Table 6, we replace the MSAM module with ordinary 3×3 convolution.

As seen from the experimental results, the CAM branch and the MSAM branch in the network structure can improve the counting accuracy in learning contextual features and acquiring accurate multi-scale features, respectively. With the MSAM branch, the counting performance of the network is obviously improved. The results prove their effectiveness.

In addition, the CMSNet we propose has a relatively small number of arguments and floating-point calculations compared to other advanced network architectures. The comparison results are shown in Table 7. The experimental performance results were obtained for the UCF-QNRF dataset, and the number of parameters and floating-point calculations were both determined based on images with an input of [1, 3, 224, 224]. From the comparison results, although our counting network is not optimal in terms of technical performance, it has much fewer parameters and floating-point computations than other advanced network structures. Therefore, in practical applications, the demand for hardware is low, and the scope of application is wide.

### 4.4. Comparison with State-of-the-Art Methods

In this section, we evaluate our approach against several approaches [2,15,16,17,41,42,48,49,50,51,52,53,54,55,56,57,58] proposed to date. The experimental results of these methods were based on the ShanghaiTech_part A&B dataset, UCF_CC_50 dataset, and UCF-QNRF dataset. During the test, each complete image in the test sets of the three datasets was directly sent to our CMSNet model. Following the standard scheme adopted in [22], we carried out 5-fold cross-validation for the UCF_CC_50 dataset. We first calculated the MAE for each test scenario and then averaged all MAEs to evaluate the performance of CMSNet in different test scenarios.

As shown in Table 8, our CMSNet achieved competitive results. Moreover, our method achieved the best experimental results on the UCF-QNRF dataset. Our method also achieved good results on the most widely used SHHA&B dataset. For the UCF_CC_50 and UCF-QNRF datasets, with high crowd density, the experimental results were more obvious. Additionally, our method achieved better performance in calculating the number for dense crowds, which is what it was designed to do. That is, the crowd features could be more effectively obtained and identified based on different head scale information in a dense crowd. Although the results of our model are not optimal on the SHHA&B datasets, our model achieves the best performance on the UCF-QNRF dataset, which has a wider range of crowd features and more complex scene changes.

## 5. Conclusions

In this paper, we propose a novel network structure named CMSNet to enhance the feature representations of the crowd in an image and improve the accuracy in obtaining information for different head scales. To this end, we propose a context-aware module (CAM) to assist the network in using surrounding information to learn the features of the crowd before and after the difference is determined. We propose a new multi-scale aggregation module (MSAM) to address the different scales of human head information in an image. By aggregating head information of different scales, the counting network can learn the feature information of different scales to calculate the number of people more accurately. Moreover, the GRF branch in the MSAM can help other multi-scale branches better learn feature information. Finally, the information from multiple modules is merged into a final density map. Extensive experiments on three challenging datasets prove that the proposed method is very competitive with the current state-of-the-art methods.

## Figures and Tables

**Table 1:** Summarizations of crowd counting datasets.

Datasets	Resolution	Number of Images	Max	Min	Total
SHHA	different	482	3139	33	241,677
SHHB	768 × 1024	716	578	9	88,488
UCF_CC_50	different	50	4543	94	63,974
UCF-QNRF	different	1535	12,865	49	1,251,642

**Table 2:** Performance comparisons of different dilation rate settings of the MSAM on SHHA.

Dilation Rates	MAE	MSE
1, 2, 3, 4	67.1	110.3
1, 2, 3, 5	66.5	113.4
1, 2, 3, 8	65.3	112.3
1, 4, 7, 9	65.5	**108.1**
**1, 2, 3, 6**	**64.9**	111.2

**Table 3:** Ablation study of the GRF on SHHA.

Model	MAE	MSE
w/o GRF	66.8	115.9
w. GRF	**64.9**	**111.2**

**Table 4:** Ablation study of the residual connection in the MSAM on SHHA.

Model	MAE	MSE
w/o residual	67.2	118.6
w. residual	**64.9**	**111.2**

**Table 5:** Ablation study of the residual connection in the CAM on SHHA.

Model	MAE	MSE
w/o residual	66.4	115.2
w. residual	**64.9**	**111.2**

**Table 6:** Ablation study of the CAM and the MSAM in the CMSM on SHHA.

Model	MAE	MSE
w/o CAM	68.2	118.8
w/o MSAM	71.0	117.6
w. CAM & MSAM	**64.9**	**111.2**

**Table 7:** Comparison of the number of parameters and floating-point calculations on the UCF-QNRF dataset.

Network Model	Year	Parameter (K)	FLOPs(G)	MAE	MSE
CSRNet [17]	2018	16,263	20.74	111.8	198.1
CAN [45]	2019	18,103	21.99	107.0	183.0
S-DCNet [46]	2019	14,979	**15.36**	104.4	176.1
ASNet [47]	2020	30,398	31.33	**91.6**	**159.7**
Ours	2022	**9668**	16.67	102.3	176.5

**Table 8:** Comparison of the different state-of-the-art methods on the SHHA, SHHB, UCF_CC_50, and UCF-QNRF datasets. The red and green numbers are the performance gains of different methods relative to CSRNet.

Network Model	Year	SHHA				SHHB				UCF_CC_50				UCF-QNRF			
		MAE		MSE		MAE		MSE		MAE		MSE		MAE		MSE	
MCNN [15]	2016	110.2	−61.6%	173.2	−50.6%	26.4	−149.1%	41.3	−158.1%	377.6	−41.9%	509.1	−28.1%	277	−130.2%	426	−104.3%
MSCNN [48]	2017	83.8	−22.9%	127.4	−10.8%	17.7	−67.0%	30.2	−88.8%	363.7	−36.7%	468.4	−17.8%	-	-	-	-
Switching-CNN [16]	2017	90.4	−32.6%	135.0	−17.4%	21.6	−103.8%	33.4	−108.8%	318.1	−19.5%	439.2	−10.5%	228	−90.0%	445	−113.4%
CSRNet [17]	2018	68.2	-	115.0	-	10.6	-	16.0	-	266.1	-	397.5	-	120.3	-	208.5	-
SANet [49]	2018	67	1.8%	104.5	9.1%	8.4	20.8%	13.6	15.0%	258.4	2.9%	334.9	15.7%	-	-	-	-
ASD [50]	2019	65.6	3.8%	98	14.8%	8.5	19.8%	13.7	14.4%	196.2	26.3%	270.9	31.8%	-	-	-	-
PACNN [51]	2019	66.3	2.8%	106.4	7.5%	8.9	19.1%	13.5	15.6%	267.9	−0.7%	357.8	10.0%	-	-	-	-
LSC-CNN [52]	2020	66.4	2.6%	117.0	−1.7%	8.1	23.6%	12.7	20.6%	225.6	15.2%	302.7	23.8%	120.5	−0.2%	218.2	−4.7%
C-CNN [53]	2020	88.1	−29.2%	141.7	−23.2%	14.9	−40.6%	22.1	−38.1%	-	-	-	-	-	-	-	-
DUBNet [54]	2020	64.6	5.3%	106.8	7.1%	**7.7**	27.4%	**12.5**	21.9%	243.8	8.4%	329.3	17.2%	105.6	12.2%	180.5	13.4%
LA-Batch [55]	2021	65.8	3.5%	103.6	9.9%	8.6	18.9%	13.6	15.0%	203	23.7%	**230.6**	42.0%	113	6.1%	210	−0.7%
MSCANet [42]	2021	66.5	2.5%	102.1	11.2%	-	-	-	-	242.8	8.8%	329.8	17.0%	104.1	13.5%	183.8	11.8%
SRNet [41]	2021	66.0	3.2%	**96.7**	15.9%	-	-	-	-	**184.1**	30.8%	232.7	41.5%	108.2	10.0%	177.5	14.9%
HADF-Crowd [2]	2021	71.1	−4.3%	111.6	3.0%	9.7	9.3%	15.7	1.9%	-	-	-	-	-	-	-	-
SRN [56]	2021	64.4	5.6%	100.2	12.9%	8.4	20.8%	13.4	16.3%	242.3	8.9%	320.4	19.4%	-	-	-	-
PDD-CNN [58]	2021	64.7	5.1%	99.1	13.8%	8.8	17.0%	14.3	10.6%	205.4	22.8%	311.7	21.6%	115.3	4.2%	190.2	8.8%
IA-MFFCN [57]	2022	**62.9**	7.8%	100.8	12.3%	9.8	7.5%	13.2	17.5%	242.7	8.8%	320.4	19.4%	-	-	-	-
Ours	2022	64.9	4.8%	111.2	3.3%	8.5	19.8%	13.3	16.8%	203.9	23.4%	259.9	34.6%	**102.3**	15.0%	**176.5**	15.3%

## Data Availability

Not applicable.
